# Beta-Eucain Versus Cocain

**Published:** 1905-01

**Authors:** 


### Beta-Eucain versus Cocain.

Otto Simon, first assistant in Prof. Czerny's surgical clinic at Heidelberg, (*Münchener Med. Wochenschrift,* Vol. 51, No. 29, July 19, 1904,) calls renewed attention to the severe intoxication which even minute quantities of cocain may produce in predisposed individuals. In a neurasthenic aged 24, death set in in two minutes after the injection into the urethra of 1¾ drams of a 1 per cent. cocain solution. Since then Simon uses exclusively beta-eucain. It has entirely the same effect as cocain when administered in 0.6 to 0.9 per cent. saline solution at the body temperature; and in 188 anesthesias a little headache and nausea was only encountered once, when the bladder was filled with 2 2-3 ozs. of a 4 per cent. solution, which vanished immediately when the bladder was washed out.

Eucain-adrenalin is especially useful in the extirpation of subcutaneous or deep-seated tumors, lipomata, mammary adenomata and strumous lymphatic glands, for excision of tumors, cancers and angiomata, and for minor operations around the head and face. For the extirpation of tubercular glands and for Winkelmann's hydrocele operation, it may be used alone or with adrenalin. In chronic tubercular osteitis, ganglion, for the removal of foreign bodies, for tendon suture, and in operating ingrown toenails, eucain should be used alone; and he also employed it for cystoscopies and litholapaxies.

For infiltration anesthesia he advises a warm 0.5 per cent to 1 per cent. eucain solution with 0.6 per cent. to 9 per cent. sodium chloride and 1:20,000 to 1:30,000 adrenalin. Adrenalin strengthens the eucain anesthesia and has no undesirable effect in these dilutions. Where applicable, Oberst's method, with 1 per cent. beta-eucain, is preferable to simple eucain infiltration and even to eucain-adrenalin.

